# Microsatellite typing and susceptibilities of serial *Cryptococcus neoformans *isolates from Cuban patients with recurrent cryptococcal meningitis

**DOI:** 10.1186/1471-2334-10-289

**Published:** 2010-10-04

**Authors:** María T Illnait-Zaragozí, Gerardo F Martínez-Machín, Carlos M Fernández-Andreu, Ferry Hagen, Teun Boekhout, Corné HW Klaassen, Jacques F Meis

**Affiliations:** 1 Department of Bacteriology and Mycology, Instituto Pedro Kourí, Havana, Cuba; 2 CBS-Fungal Biodiversity Centre, Utrecht, The Netherlands; 3 Department of Medical Microbiology and Infectious Diseases, Canisius Wilhelmina Hospital, Nijmegen, The Netherlands

## Abstract

**Background:**

*Cryptococcus neoformans *is commonly associated with meningoencephalitis in immunocompromised patients and occasionally in apparently healthy individuals. Recurrence of infection after initial treatment is not uncommon. We studied *C. neoformans *isolates from 7 Cuban patients with recurrent cryptococcal meningitis. Antifungal susceptibility and genotyping with microsatellite molecular typing were carried out.

**Methods:**

Isolates (n = 19) were recovered from cerebrospinal fluid, blood, urine and semen. Antifungal susceptibilities for amphotericin B, fluconazole, flucytosine, itraconazole, voriconazole, posaconazole and isavuconazole were tested by CLSI M27A3 broth microdilution method. Genotyping was done using a panel of 9 microsatellite (STR) markers: (CT)n, (TG)n, (TA)n, (CTA)n, (TCT)n, (CCA)n, (TTAT)n, (ATCC)n and (TATT)n.

**Results:**

The average number of isolates/patient was 2.71. The mean time interval between the collection of any two isolates was 52.5 days. All strains were identified as *C. neoformans *var. *grubii *(serotype Aα). Although none of the strains were resistant to the studied drugs, in serial isolates from two patients, MICs values of triazoles increased 4-5 log_2 _dilutions over time. STR patterns showed 14 distinctive profiles. In three patients the recurrent infection was associated with genotypically identical isolates. The four other patients had relapse isolates which were genotypically different from the initial infecting strain.

**Conclusion:**

Recurrences of cryptococcal meningitis in our series of patients was not associated with development of drug resistance of the original strain but by an initial infection with different strains or a reinfection with a new strain.

## Background

The incidence of cryptococcosis started to increase with the beginning of the acquired immune deficiency syndrome (AIDS) epidemic in the early 1980 s. Its frequency declined in the Western world since the mid 1990 s due to the use of highly active antiretroviral therapy (HAART). However, cryptococcal meningitis is still one of the most common life-threatening opportunistic fungal infections in immunocompromised patients, particularly among those with AIDS in Sub-Saharan Africa [1] and Asia [2]. These patients show a high tendency to relapse despite effective antifungal therapy. Early studies performed with karyotyping and restriction fragment length polymorphism (RFLP) analysis of serial isolates of AIDS patients in New York concluded that recurrent infection was caused through persistence of the original strain and not from infection with new strains [3,4]. A later study using the same typing technique but with isolates from AIDS patients from Uganda found that among 17 patients with more than 1 cerebrospinal fluid (CSF) isolate of *Cryptococcus neoformans*, sequential isolates were identical or highly related in 12 patients [5].

Several treatment strategies have been used in patients with cryptococcal meningitis but the optimum regimen is still not clear. Amphotericin B with or without flucytosine remain the agents of choice for induction therapy while fluconazole has proven to be superior for long-term maintenance therapy [6,7]. High dose fluconazole with flucytosine is an oral treatment alternative although this regimen is not as effective as amphotericin B and flucytosine [8]. Different molecular typing methods have been used in the epidemiological analyses of clinical and/or environmental isolates of *C. neoformans*, including electrophoretic karyotyping, PCR fingerprinting, random amplified polymorphic DNA analysis, RFLP analysis, MLST and amplified fragment length polymorphism analysis with divergent results [9-11]. To determine whether recurrences of cryptococcal meningitis in seven Cuban patients were due to development of drug resistance or to infection by multiple strains, we tested the *in vitro *antifungal susceptibility and determined the genotypes of the sequential isolates using a recently described microsatellite based assay [12].

## Methods

### Fungal isolates

From the stock collection of the mycology laboratory at the Instituto de Medicina Tropical "Pedro Kourí" (IPK) in Havana, Cuba, 19 clinical isolates of *C. neoformans *from patients with recurrent infection were selected for this study. The isolates were recovered at different time intervals from cerebrospinal fluid (n = 16), blood, urine and semen (one each) from 7 patients (6 HIV positive and 1 HIV negative) admitted at the institute between 1995 and 2001, just before HAART was introduced in Cuba (Table 1). All patients were admitted to the clinical AIDS care division at the IPK. Initial isolates were obtained at diagnosis and before any antifungal therapy was used and follow up isolates were from patients during or after treatment with antifungal drugs. Repeat lumbar puncture was only performed in those cases which did not react within two weeks of therapy or those who showed a clinical deteriorisation during or after treatment. From each culture positive sample a single isolate from morphological similar colonies was archived. Species identification was initially done by growth on canavanine-glycine-bromothymol blue (CGB) agar and strains were stored in sterile water at room temperature until the study was carried out. From initial isolation until the study the isolates were typically subcultured between 4 and 6 times. Before use, the identification of all isolates was repeated with a commercial identification system (Auxacolor 2; Bio-Rad, Marnes-la-Coquette, France).

**Table 1:** Clinical data, origin and date of isolation of the studied strains.

Patient **Nr**.	Sex	HIV/ Year of diagnosis	Sample	Isolation date	Interval between each isolate in days (number)	Total of days	Antifungal treatment
1	Male	+/1991	Blood	14/04/95	0 (08-36-09-92)	0	AmB+Flu Flu (maintenance)
			
			CSF	14/04/95	0 (08-36-09-75)	0	AmB+Flu Flu (maintenance)
			
			CSF	18/07/95	96 (08-36-10-01)	96	AmB+Flu

2	Male	+/1990	CSF	05/09/95	0 (08-36-09-97)	0	Flu+Itr
			
			Urine	08/12/95	95 (08-36-09-70)	95	Flu
			
			CSF	10/01/96	34 (08-36-09-91)	129	Flu

3	Female	+/1994	CSF	18/01/96	0 (08-36-09-71)	0	AmB+Flu Flu (maintenance)
			
			CSF	29/02/96	34 (08-36-09-72)	34	AmB+Flu

4	Male	+/1995	CSF	23/04/97	0 (08-36-09-82)	0	AmB+FC+Flu
			
			CSF	06/05/97	14 (08-36-09-89)	14	AmB+FC+Flu

5	Male	+/1998	CSF	15/04/00	0 (08-36-09-24)	0	AmB Flu (maintenance)
			
			CSF	26/10/00	194 (08-36-09-25)	194	AmB+FC

6	Female	+/1996	CSF	12/06/00	0 (08-36-10-56)	0	AmB+FC Flu (maintenance)
			
			CSF	12/10/00	123 (08-36-10-52)	123	AmB Flu (maintenance)
			
			CSF	30/10/00	19 (08-36-10-53)	142	AmB+FC

7	Male	-	CSF	09/01/01	0 (08-36-09-26)	0	AmB+Itr Flu (maintenance)
			
			CSF	17/04/01	99 (08-36-09-30)	99	AmB+Flu Flu (maintenance)
			
			CSF	07/06/01	52 (08-36-09-78)	151	Flu+Itr
			Semen	19/06/01	13 (08-36-09-42)	164	Liposomal AmB

### Antifungal agents and susceptibility testing

Broth microdilution testing was performed in accordance with Clinical and Laboratory Standards Institute document M27-A3 guidelines [13]. Standard antifungal powders of amphotericin B (Sigma, The Netherlands), flucytosine (Valeant Pharmaceuticals, The Netherlands), fluconazole (Pfizer Central Research, U.K.), itraconazole (Janssen-Cilag, The Netherlands), voriconazole (Pfizer Central Research, U.K.), posaconazole (Schering Plough, USA), and isavuconazole (Basilea Pharmaceutica, Switzerland) were used. The stock solutions of the drugs were prepared in the appropriate solvent. The final concentrations of the antifungal agents were 0.016 to 8 μg/mL for amphotericin B, itraconazole, voriconazole, and posaconazole; 0.063 to 32 μg/mL for flucytosine and fluconazole; and 0.004 to 4.00 μg/mL for isavuconazole. After 72 h incubation at 35°C the minimum inhibitory concentration (MIC) was defined as the lowest concentration of drug showing absence of growth for amphotericin B and a prominent reduction of growth (≥ 50%) for the other antifungal agents compared to the drug-free growth control. The MICs were read optically and spectrophotometrically at 420 nm after agitation. *Candida parapsilosis *ATCC 22019 and *C. krusei *ATCC 6258 were used as quality control [13].

### Mating-, sero- and genotyping

*C. neoformans *isolates were grown on Sabouraud's dextrose agar at 30°C for 48 h and DNA was obtained from freshly grown cells using a MagNA lyser/MagNA Pure protocol (Roche Diagnostics, Almere, the Netherlands). The mating- and serotype was determined using four different PCRs that specifically amplify the mating-type **a **or α allele of the *STE20 *locus for either serotype A or D isolates [14]. The reference isolates CBS9172 (**a**A), CBS8710 (αA), CBS10511 (**a**D) and CBS10513 (αD) were included as positive control for each of the four PCRs.

STR analysis was performed in two steps as described previously [12]: i) Amplification of STR loci by PCR: three separate multiplex PCRs were used (CNA2, CNA3, and CNA4, respectively), each amplifying three different STRs. For every multiplex PCR, one of the amplification primers was labelled with carboxyfluorescein (FAM), hexachlorofluorescein (HEX), or tetrachlorofluorescein (TET) at the 5' end, respectively. In addition to the amplification primers (concentrations according to reference [11]), each PCR mixture contained 0.2 mM deoxynucleoside triphosphates, 1 U of FastStart Taq DNA polymerase (Roche Diagnostics), 2 mM MgCl_2 _and 1 ng of genomic DNA in 1 × reaction buffer. Thermocycling was performed in a T1 thermocycler (Biometra, Göttingen, Germany) by using the following thermal protocol: 10 min of denaturation at 95°C, followed by 35 cycles of 30 s of denaturation at 95°C, 30 s of annealing at 60°C, and 1 min of extension at 72°C. Before the reaction mixtures were cooled to room temperature, an additional incubation for 10 min at 72°C was performed. All temperature transitions were performed with maximal heating and cooling settings (5°C/s). ii) Detection and sizing of amplification products with subsequent assignment of repeat numbers: the fragments obtained were combined with the ET550-R size standard (GE Healthcare, Diegem, Belgium) and analyzed on a MegaBACE 500 automated DNA platform (GE Healthcare), according to the instructions of the manufacturer. Electropherograms were analyzed using Fragment Profiler 1.2 software (GE Healthcare). Identical isolates were those that possessed alleles with the same number of repeat units in all nine loci. Consistent with the previous separation of genotypes into microsatellites complexes (MC's), isolates with genotypes that differed in up to two loci were considered to be genetically related [12]. Genotypes differing in more than two loci were considered to be unrelated. The study was approved by the Scientific Council and Ethics Committee of the Instituto Pedro Kouri, Havana, Cuba.

## Results

The majority of strains were obtained from HIV positive patients, except those recovered from patient 7 for whom no underlying disease could be demonstrated (Table 1). The mean number of isolates/patient was 2.71 (range 2-4 isolates/patients). The mean time between collection of any two isolates was 52.5 days (range 13-123 days). All patients received antifungal treatment but only the HIV negative patient (nr. 7) survived. All clinical strains were identified as *C. neoformans *var. *grubii *serotype A and mating-type α.

For the antifungal susceptibility testing, no differences between visual and spectrophotometric readings were observed, and the MICs for the quality control strains were all within the suggested reference ranges (data not shown). Table 2 summarizes the *in vitro *susceptibilities of the isolates according to the origin of the strains. When all data were considered together, the widest ranges and highest MICs were for fluconazole (0.25 to 8 μg/mL) and flucytosine (0.5 to 8 μg/mL) and the lowest were for isavuconazole, voriconazole, and posaconazole. Amphotericin B, posaconazole and isavuconazole exhibited similar MICs patterns among all the studied isolates. Isolates from patients 6 and 7 exhibited a stepwise increase among serial isolates for some of the drugs. Increased MICs values of at least 4 log_2 _dilutions over the time for fluconazole, itraconazole, voriconazole and isavuconazole were observed in patient 6. In patient 7 higher MICs were found between initial and last isolate for fluconazole (4 log_2_), itraconazole (6 log_2_) and voriconazole (5 log_2_). Amphotericin B, flucytosine and posaconazole demonstrated maximally only a 1-3 log_2 _difference among serial isolates.

**Table 2:** Minimum inhibitory concentration of all *C. neoformans *var. *grubii *isolates for seven antifungal drugs

**Patient Nr**.	Strain **Nr**.	Minimum Inhibitory Concentration (μg/mL) reading at 72 h						
		
		AmB	FC	Flu	Itr	Vor	Pos	Isa
1	08-36-09-92	0.125	8	4	0.125	0.016	0.125	0.016
	
	08-36-09-75	0.25	2	4	0.063	< 0.016	0.063	< 0.004
	
	08-36-10-01	0.25	4	4	0.25	0.125	0.125	< 0.004

2	08-36-09-97	0.125	2	1	0.031	0.063	0.125	< 0.004
	
	08-36-09-70	0.25	2	2	0.063	0.063	0.125	0.016
	
	08-36-09-91	0.125	1	1	0.031	< 0.016	0.063	0.008

3	08-36-09-71	0.25	2	2	0.063	0.063	0.016	0.016
	
	08-36-09-72	0.125	8	2	0.125	0.125	0.031	< 0.004

4	08-36-09-82	0.25	2	2	0.031	0.031	0.031	0.008
	
	08-36-09-89	0.25	1	4	0.031	0.125	0.063	0.008

5	08-36-09-24	0.25	2	1	< 0.016	< 0.016	0.016	0.004
	
	08-36-09-25	0.25	1	0.25	0.031	< 0.016	0.016	< 0.004

6	08-36-10-56	0.125	0.5	0.25	< 0.016	< 0.016	0.063	< 0.004
	
	08-36-10-52	0.125	0.5	4	0.125	0.125	0.016	0.031
	
	08-36-10-53	0.25	1	8	0.125	0.25	0.016	0.063

7	08-36-09-26	0.25	0.5	0.5	< 0.016	< 0.016	0.031	0.016
	
	08-36-09-30	0.25	0.5	0.5	0.031	0.031	0.016	0.004
	
	08-36-09-78	0.125	1	1	0.031	0.063	0.016	< 0.004
	
	08-36-09-42	0.25	4	8	0.5	0.25	0.031	< 0.004

Abreviations: AmB: amphotericin B, FC; flucytosine, Flu; fluconazole, Itr; itraconazole, Vor; voriconazole, Pos; posaconazole, Isa; isavuconazole

**Figure 1:**
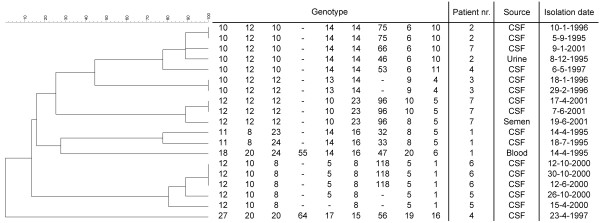
**Details of the 19 *C. neoformans *isolates from 7 patients and relationship between the obtained genotypes**. The numbers below the genotype correspond to the number of repetitions observed in markers CNA2a, CNA2b, CNA2c, CNA3a, CNA3b, CNA3c, CNA4a, CNA4b and CNA4c, respectively. A hyphen indicates that no result was obtained. The dendrogram is based on a categorical analysis using UPGMA clustering. The scale bar indicates the percentage similarity.

STR patterns of the 19 isolates showed 14 distinct profiles. Three patients (patients 2, 3 and 6) had genotypically identical isolates over the course of time. The serial isolates from patient 6 were genotypically identical but also showed the largest difference in MIC for the triazoles including drugs which have not been used (itraconazole, voriconazole, isavuconazole). The other four patients were probably infected by more than one genotype (Figure 1) although this might be biased because from each positive CSF culture only one colony was archived for future study.

## Discussion

No previous studies of the antifungal susceptibility and genetic diversity of sequential isolates of *C. neoformans *obtained from individual patients have been done in Cuba. Here we studied 19 serial clinical strains of *C. neoformans *from 7 patients with recurrent cryptococcal meningitis during the pre-HAART period by analyzing their antifungal susceptibility and molecular profiles.

Our results on the *in vitro *activities of the main antifungal drugs are similar to those published previously [15-17]. Amphotericin B has long successfully been used to treat various yeasts and mould infections. In this series there was only one log_2 _dilution difference between the sequential isolates with a highest observed MIC of 0.25 μg/mL way below the suggested breakpoint for resistance of MIC ≥2 μg/mL, which was found to be associated with therapeutic failure [18]. All flucytosine MICs were < 16 μg/mL which are regarded as susceptible [7,14]. Among the azoles, fluconazole showed the lowest *in vitro *activity. In fact, previous reports have already demonstrated the low activity of this drug against *C. neoformans *isolates, even though it has proven to be more active *in vivo *[19]. According to these authors, the good therapeutic results obtained are largely attributable to its high concentrations in cerebrospinal fluid. Although no resistance has been found in the present collection of isolates (MIC < 16 μg/mL) [20], a stepwise increase of 4 and 5 dilutions was found in 2 patients suggesting development of reduced levels of antifungal susceptibility[7]. In one patient (nr. 6) the sequential isolates were genotypically identical. Of interest is the finding that the increase of fluconazole MICs over time in patients 6 and 7 parallels data with itraconazole, voriconazole and isavuconazole but not with posaconazole. These observations support previous work that suggests the development of cross-resistance of fluconazole with other triazoles [20]. Isavuconazole is an experimental broad-spectrum antifungal triazole active against clinically relevant yeasts and moulds [21]. Our results are in agreement with previously published studies which demonstrated a high *in vitro *activity of this drug for *C. neoformans *[17,22]. It has been demonstrated that there are no trends towards higher MICs for strains isolated from patients who failed to respond to a given therapy compared to isolates from patients who did not [23]. On the other hand, patient 4 was infected with two different genotypes with similar susceptibility profiles. Because the short time in between each isolation it is suggestive that this was not a recurrent infection, it is possible that the patient was infected simultaneously with both strains. Patients 2 and 5 had three and two isolates respectively with similar susceptibility patterns. In the first case only the CSF isolates were genotypically similar. This might imply that this patient was also infected simultaneously with more than one strain however there might be a bias because mixed infections in one sample of morphologically similar cryptococci might have been missed. In the second case, isolates differed in one marker and were thus considered to be genetically related. These observations suggest microevolution of *C. neoformans *during human infection. This process may allow the fungal population to change and escape eradication by the immune system, and thus cause chronic infections as suggested by Jain *et al*. [24]. Patient 1 had two baseline isolates, from blood and CSF, that had similar susceptibility but were genotypically different. Since both genotypes were isolated simultaneously this could mean that the patient was infected with both strains at time of first sampling. As has been stated before we might have missed mixed infection at baseline because not all (morphologically) similar colonies were studied. After more than three months of fluconazole maintenance therapy, the patient was re-admitted because signs and symptoms of relapse. The CSF isolate obtained at that time had higher MIC values for voriconazole and a different genotype compared with the previous isolates. These findings suggest two possibilities: i) the patient was re-infected with a new strain during the maintenance therapy or ii) the initial strains underwent genetic microevolution. Cerebrospinal fluid from patient 7 remained microscopically (Indian ink) and culture positive during more than 5 months, despite antifungal therapy. Only the second and third isolate showed the same genotype which was different from the first and the fourth isolate. This could be explained by simultaneous infection with more than one strain or by re-infection with a new strain during treatment which developed genetic changes over time. This is corroborated by the increased MICs values for fluconazole, itraconazole and voriconazole of the last isolate.

Other authors have studied the molecular relationship of *C. neoformans *isolates obtained from the same episode of infection or during a recurrent infection with several different techniques [10,11,24-26]. Although the obtained results are variable, most authors suggest that persistence or recurrence of the infection is caused by relapse rather than re-infection and/or microevolution of the original isolate [20,25-28]. Multiple strain infection was rarely considered until a recent study reported a high frequency of mixed infections in 20% of patients with cryptococcal disease and speculated that multiple strains could be exogenously acquired from the environment, either simultaneous or sequentially [29]. A bias was entered in our study because only one colony was selected for archiving from each sampling point (because all growing colonies were morphologically similar). Morphological similar colonies might have different genotypic backgrounds as has been shown recently [29].

## Conclusions

STR typing as presented in this study has not been largely used for *Cryptococcus *molecular characterization. This new typing technique allowed the observation of high genetic variability among the studied clinical isolates keeping in mind the previous mentioned limitation of this study and we confirm the observation of within-host strain diversity [29]. Recurrence of infection in the majority of these patients were not associated with drug resistance but probably by co-infection with different strains or strains genetically modified during the long maintenance therapy.

## Competing interests

JFM received grants form Astellas, Basilea, Merck, and Schering-Plough. He has been a consultant to Basilea, Merck and Schering-Plough and received speakers fees from Gilead, Janssen Pharmaceutica, Merck, Pfizer, and Schering-Plough. CHWK received a grant from Pfizer. All other authors: no potential conflicts of interest.

## Authors' contributions

MTIZ and FH carried out the susceptibility testing and molecular typing, and drafted the manuscript. MTIZ GFMM and CMFA participated in the design of the study, contributed materials and cared for the clinical management of the patients. TB CHWK and JFM conceived of the study, and participated in its design and coordination and helped to draft the manuscript. All authors read and approved the final manuscript.

## Pre-publication history

The pre-publication history for this paper can be accessed here:

http://www.biomedcentral.com/1471-2334/10/289/prepub
